# Impacts of Food‐Based Flock Size on Foraging Patterns, Activity Time Budget and Foraging Efficiency: Flexible Behavioral Responses of the Wintering Oriental Storks (*Ciconia boyciana*) to Changes in Aquaculture at Shengjin Lake, China

**DOI:** 10.1002/ece3.71037

**Published:** 2025-02-27

**Authors:** Lei Cheng, Lizhi Zhou, Chao Yu, Yiwei Bao

**Affiliations:** ^1^ School of Resources and Environmental Engineering Anhui University Hefei People's Republic of China; ^2^ School of Biological Engineering Huainan Normal University Huainan People's Republic of China; ^3^ Anhui Province Key Laboratory of Wetland Ecosystem Protection and Restoration (Anhui University) Hefei People's Republic of China; ^4^ Anhui Shengjin Lake Wetland Ecology National Long‐term Scientific Research Base Dongzhi People's Republic of China; ^5^ College of Life and Environment Science Huangshan University Huangshan People's Republic of China

**Keywords:** activity budget, flock size, food‐based, foraging strategy, Oriental Stork

## Abstract

Food resources, as key limiting factors for wintering waterbirds, influence their habitat selection patterns and foraging behaviors. Meanwhile, seasonal fluctuations in water levels and human exploitation of lake wetlands both affect the availability of food. Therefore, knowledge of the spatio‐temporal dynamics of habitat utilization and adaptive behavior strategies can provide insights into how animals adapt to habitat changes in wetlands and has important conservation implications. In this study, we examined the effect of dynamic food resource supply on the spatial patterns, activity budget, and foraging strategy of Oriental Storks ( *Ciconia boyciana* ) at Shengjin Lake in China during a period where extensive fishing nets were present limiting the movement and dispersal of waterbirds (“pen culture period, PP” in 2017 winter) and a period after the removal of these pens during wetland restoration (“non‐pen culture period, NPP” in 2018 winter). In comparing with the wintering storks in NPP, we demonstrated an overall loss of range and a significant reduction in population size in PP, which were probably due to habitat alteration and fragmentation triggered by pen culture. We reported that a higher overall time budget in foraging and locomotion and a comparatively lower in other behaviors with storks in PP. Net pens resulted in limited activity areas of the storks and a reduction in food availability due to habitat alteration and fragmentation, thus resulting in a more flexible and radical trend in the foraging patterns of the wintering storks was triggered by the combined effects of the net pens removal and habitat connectivity in NPP. Pen culture had resulted in a more conservative foraging strategy and a homogenization of behavioral composition for wintering storks at the lake. Our study highlighted the behavior‐based results may provide key information to conceive management and conservation plans for wintering waterbirds.

## Graphical abstract

This paper provided a small‐scale but important case study, adding to our knowledge of Oriental Storks in aquaculture fisheries in the middle and lower Yangtze River floodplain, and analyzed the impacts of food‐underpinned flock size on activity time budget adjustments and foraging strategy adaptation of the wintering Oriental Storks. The results have implications for the conservation management of other endangered waterbirds facing pressures from culture‐based fisheries.

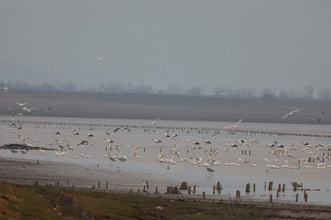

## 1 Introduction

Many animal species live in groups for either a part of or for the entirety of their annual life cycle (Krause and Ruxton 2002). The ready availability of information from conspecifics allows animals to make better decisions about habitat selection, enhancing foraging rate, avoiding predators, and sharing social information (Evans et al. 2016; Gil et al. 2018; Morinay et al. 2023). Furthermore, living in groups can help individuals to find food easily (Krause and Ruxton 2002), increase food‐finding efficiency (Evans et al. 2016), and lower predation risk (Eccard et al. 2020). As individuals within a group, such as wintering waterbirds, animals adjust their behavior budgets (Bensizerara and Chenchouni 2019; Mukherjee et al. 2023) and foraging strategies (Kuwae et al. 2010; Lunardi and Macedo 2014) in response to habitat dynamics. These adjustments help them cope with environmental factors like human disturbances (e.g., wetland reclamation and damming) (Fang et al. 2005; Yu et al. 2017; Wang et al. 2020) and natural hydrological fluctuations (e.g., water level and rate of change) (Li et al. 2019). In the flood pulse concept, food availability is linked to the inundation of the floodplain; fishes have a high aggregation degree and availability to catch when water levels are low (Linhoss et al. 2012). When habitat changes, the spatial distribution and flock size of waterbirds will be affected directly or indirectly by the distribution and availability of food resources (Wang et al. 2020). In turn, this will also lead to reallocation of time budgets to adapt to new habitat conditions and obtain maximum fitness (Czech and Parsons 2002; Wood et al. 2010). Hence, quantifying activity time budget patterns and investigating how animals balance the trade‐off between crucial time budget compositions can help us to understand how animals living in groups adapt to their habitat changes.

**FIGURE 1:**
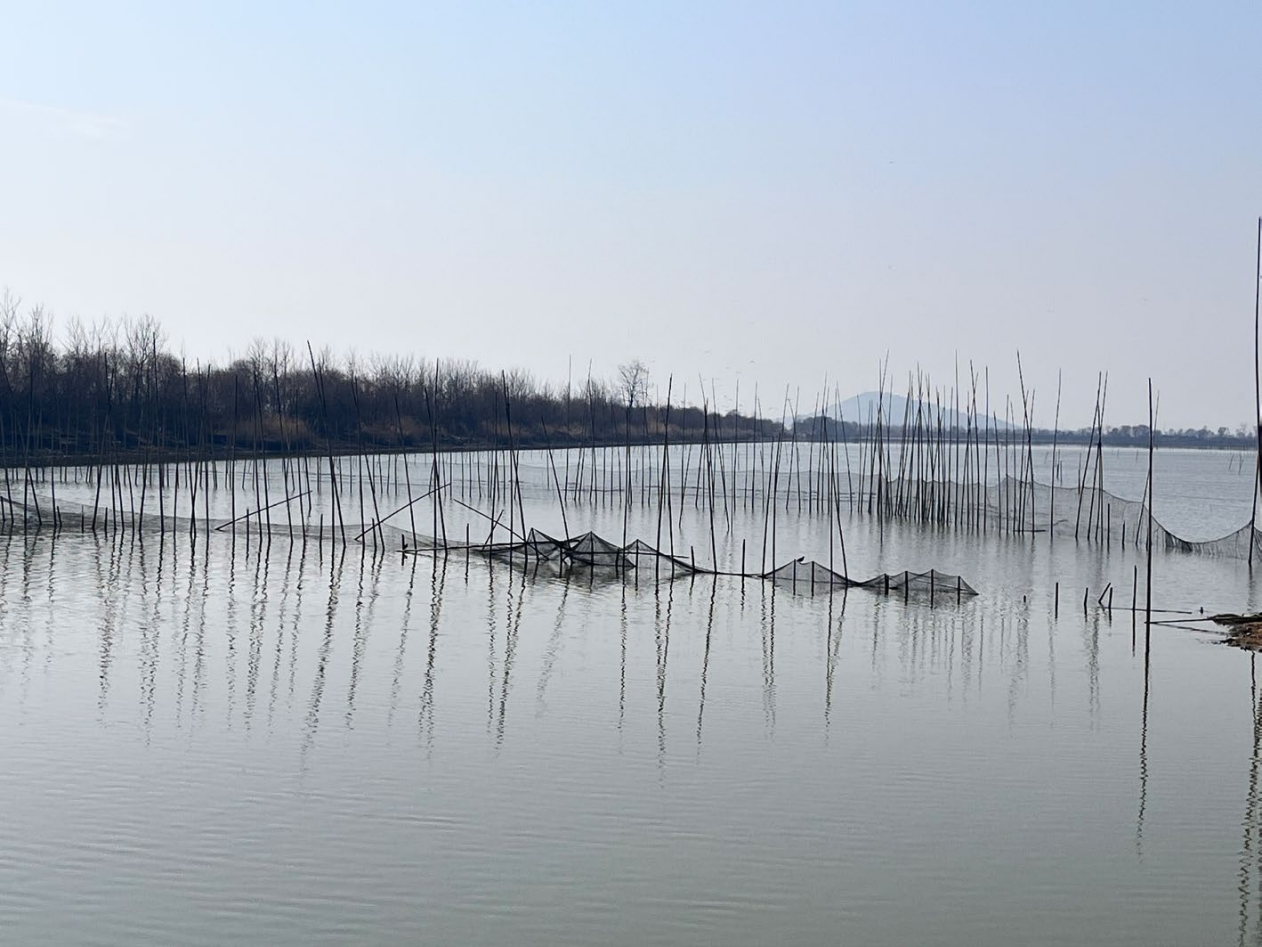
Pen cultures in the lakes of the middle and lower Yangtze River floodplain in 2017.

The middle and lower Yangtze River floodplain provides the waterbirds with suitable foraging habitats during winter seasons (Zhang et al. 2022). Due to the seasonal water level fluctuations and human disturbance in the river‐connected lakes, the distribution and behavior of wintering waterbirds are highly associated with habitat and food availability (Wang et al. 2013; Li et al. 2015). During the winter, the low water level makes large areas of submerged lakeshore exposed and attracts large numbers of waterbirds to gather here. As such, the change in water level requires waterbirds to adjust their activity time budgets for better foraging. Fishery aquaculture brings huge economic benefits. Meanwhile, the loss of natural wetlands has led to a reduction in suitable habitats and food resources for waterbirds. In the process, aquaculture can provide an abundant food supply for fish‐eating waterbirds and play an important role in supplementing their nutritional requirements. Pen culture is typically constructed from meshy materials supported by bamboo frameworks, which effectively confine cultured fish within the designated aquatic enclosure (Figure 1). However, net pens divide the natural lakes into discrete sections, which result in habitat fragmentation (Wu et al. 2001). Therefore, waterbirds had to adjust their activity budget patterns and foraging strategies to respond positively.

Oriental Stork ( *Ciconia boyciana* ) is a large migratory colonial wading waterbird, which is an endangered (EN) species in the IUCN Red List of Threatened Species (BirdLife International 2018) and the first category of the nationally protected wild species in China. The global population that winters in China is mainly limited to the lakes situated in the middle and lower Yangtze River floodplain (Zhou et al. 2013; Cheng et al. 2023). The main food resources for storks are fishes, which changes in habitat conditions can also affect the abundance and distribution of storks (Jiang et al. 2019; Wang et al. 2019). Therefore, net pens and water level fluctuations directly affect fish, the food sources of storks, and in turn affect the spatial distribution and flock size. However, little is known about how the Oriental Storks adjust their behavioral patterns and foraging strategies to respond to changes associated with disturbed and natural conditions of external environmental factors, which are important aspects of life history and ecological adaptations that should be of concern in future conservation plans.

The main goal of this study is to examine the effect of flock size and spatio‐temporal changes driven by food resources on the foraging patterns and the activity time budgets of wintering storks at Shengjin Lake. To this end, we evaluated the effectiveness of wetland restoration (through removing net pens) by comparing waterbird distribution and behavior between pen culture period (PP, 2017 winter) and non‐pen culture period (NPP, 2018 winter). We aimed to shed light on how storks allocate their activity time budgets at the collective level as a response to habitat changes. Based on the results of previous studies, the following three hypotheses were tested: (a) the time budget of foraging and locomotion would increase with the flock size of storks; (b) the spatial distribution and flock sizes of storks were significantly larger and concentrated after the net pens were removed; (c) storks reduce the foraging rate to increase the time gap to search for food in small flock sizes by visual techniques and increase the rate of foraging to increase the chances of food by tactile techniques when in large flock sizes. Based on our study, we can provide suggestions for the future conservation and habitat management of Oriental Storks in the wintering grounds.

## 2 Methods

### 2.1 Study Area and Species

**FIGURE 2:**
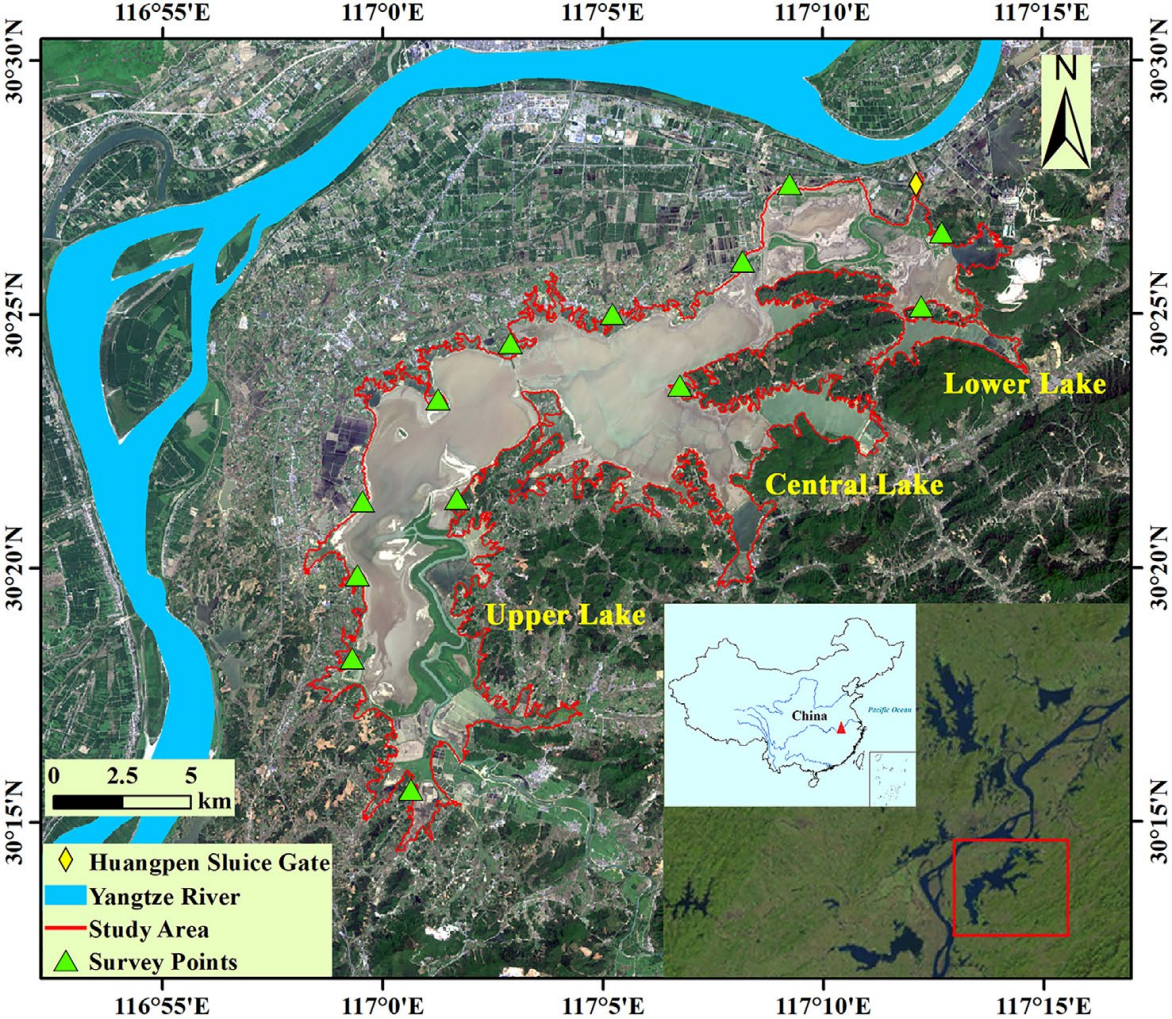
The study area and observed sites for wintering Oriental Storks at Shengjin Lake.

Shengjin Lake (30°15′‐30°30′ N, 116°55′‐117°15′ E) is part of the lake‐river complex ecosystem of the Yangtze River floodplain (Figure 2). It is an important stopover and wintering site for migratory waterbirds on the East Asian‐Australasian flyway. The lake is in the north subtropical humid monsoon climate, with an average annual temperature of 16.1°C and average annual rainfall of 1600 mm (Li et al. 2019; Song and Zhou 2019). Zhangxi and Tangtian Rivers are the two main tributaries of Shengjin Lake and run into the upper and central lakes, respectively. Huangpen Sluice is located in the lower lake, controlling the water level fluctuations of Shengjin Lake. Shengjin Lake has significant hydrological fluctuations with a mean water level of the lake at 8.9 m in winter and 12.5 m in summer (Huanghai elevation) (Li et al. 2019). The surface area of the lake varies between 13,000 hm^2^ during high water level periods and 3300 hm^2^ during low water level periods (Song 2019; Song and Zhou 2019).

Since the Huangpen Sluice had been established, fishery declined significantly, later accompanied by the fish migration passage of the Yangtze River–Shengjin Lake being disrupted. In 1995, aquaculture at Shengjin Lake shifted from natural fishing to managed stock‐enhanced fish resources (pen culture), which led to an increase in fishery production (Song 2019). A large area of the freshwater fishery with herbivorous fishes and mitten crabs as the main products occurred at Shengjin Lake thereafter. The fishery achieved a relatively high production in the first few years but then declined significantly again as macrophytes reduced. By late 2017, the net pens area at Shengjin Lake had reached 64 ha, which is mainly distributed in the middle and lower lakes (Song 2019). Net pens divided the natural lakes into discrete sections, not only blocking the connectivity of the lacustrine habitats but also limiting the movement and dispersal of waterbirds. Since 2018, the Shengjin Lake National Nature Reserve Management Bureau has removed all net pens (cumulative length of 750,000 m) and completely stopped artificial fishery farming activities in the lake (Song 2019).

The forage habitats of the wintering Oriental Storks are mainly located in the upper and lower parts of Shengjin Lake, where most of the storks gather and abundant fishery resources are available to support them. Wangba, Landaochen, Lianhe, Chi'an, Yang'etou, Yanwo, and Waipai are the main foraging sites. At Shengjin Lake, the main food sources for the storks are fishes, such as *Cyprinus carpio* , *Carassius auratus* , *Pelteobagrus nitidus*, *Monopterus albus* , and other aquatic life (*Cipango paludina sinensis*, *Macrodrachium nipponensis*, *Rana nigronmaculata*, *Elaphe rufodorsata*, etc) (Wang and Yang 1995).

### 2.2 Sampling Sites and Time

We collected field data of the storks from 7:00 to 17:00 during two successive winters from November to March (2017 winter and 2018 winter). We observed the storks along the lake bank at representative points (Wangba, Landaochen, Lianhe, Chi'an, Yang'etou, Yanwo, and Waipai) that covered nearly the same observation areas as Wang and Yang (1995) and added some new survey points (Jiangba, Longjiazui, Babaizhang, and Jiangchong) according to field observations (Figure 2). To avoid double counting, distances between any two survey points are > 1 km (Li et al. 2019). These sampling sites were observed to have large numbers of wintering storks at Shengjin Lake, and the flock size was large and stable.

### 2.3 Measuring Flock Size

We divided the wintering season for storks at Shengjin Lake into three periods, that is, early (October–December), middle (January–February), and late (March–April) based on the seasonal water level fluctuation and migration phenology of the storks. To identify the main habitat types used by storks and their spatial distribution during different wintering periods, we conducted three surveys each month with early, mid, and late at Shengjin Lake from October 2017 to April 2018 and October 2018 to April 2019. At each sampling site, the skilled bird observers used the “look‐see” counting method to estimate flock size on sunny days without strong wind or heavy fog (Delany 2005). Storks within a radius of approximately 1 km of each point were recorded using monocular (SWAROVSKI 20‐60 × 85, Absam, Austria) and binocular telescope (SWAROVSKI 8 × 42 WB, Absam, Austria). Specifically, the individual counting method was applied whenever the stork group was at a distance of < 200 m and the abundance did not exceed 200 individuals. When the number of individuals was > 200 individuals or if the group of birds was distant (i.e., > 200 m), the flock size was estimated using the agglomerate counting method (Zhao et al. 2019). Meanwhile, we visually estimated flock locations using premeasured distances between nearby landmarks. An individual was defined as within a flock when it was within 20 m (approximately 20 body lengths) of another individual (Zhao et al. 2019).

### 2.4 Behavioral Data Collection

Behavioral observations were performed from a relatively hidden and reasonable distance place (usually behind bushes or slopes about 200 m away from the storks), measured with a laser rangefinder (Nikon 1200S with a range of 10–1100 m). Before collecting behavioral data, we waited a few minutes for the group members to calm down and to ensure that the foraging activities of storks were proceeding without disturbances. Then we randomly selected and observed a foraging individual from the flock. A focal sampling technique comprising a ten‐min duration for the observation of individual behavior events via a monocular telescope was used (SWAROVSKI 20–60 × 85, Absam, Austria) (Martin and Bateson 1993), and behaviors were recorded on a mobile phone (storage capacity 256 G) connected to the monocular telescope through a converter (50–65 MM). After recording a full 10 min, we will proceed to select another foraging individual from the flock for behavioral sampling. If we lost sight of the focal individual within 10 min, the sampe was abandoned. Before each behavioral sampling, we recorded the date, time, site, flock size, habitat type, and water level (the data from the hydrological information network of Anhui Province: http://yc.wswj.net/ahsxx/LOL/?refer=upl&to=public_public).

Based on our field observations and published literature, we defined the following behaviors of the storks: foraging (rapid pecking of the beak toward the water), alert (neck stretched vertically with its head horizontal and eyes scanning the surrounding), resting (standing on the ground with alone or two feet and keeping still), locomotion (an act of moving from place to place), maintenance (e.g., preening or flapping the wing or shaking the feather), and social behavior (e.g., chasing or threatening or attacking between interspecies and intraspecies) (Ma et al. 2006). For each focal individual, we recorded the time spent performing foraging behaviors, the time spent on alert, and the time spent on other behaviors (locomotion, resting, maintenance, and social).

Furthermore, we focused on studying the foraging behavior of Oriental Storks, specifically their behavior in searching for food, handling food, and swallowing. In this process, we made a record of the following parameters: starting and ending time of each foraging bout, number of pecking, number of fish caught, number of other waders foraging in the same area, and the mode of food capture (tactile/visual). We quantified the water depth at which the Oriental Stork inhabits by observing the position where the leg reaches the water surface, using the tibiotarsus‐tarsometatarsus joint as the reference for division. Meanwhile, we determined the fish size by observing the moment when the Oriental Stork swallows the fish, with the length of its beak serving as the reference. In the later data processing, we play backed the valid audio data and obtained the specific details of the foraging behavior.

### 2.5 Statistic Methods

To investigate whether significant changes in population numbers and distribution had occurred at Shengjin Lake, we chose the abundance of Oriental Storks as the dependent variable and pen culture as the independent variable. The average number of wintering storks in the lake was used to evaluate the importance of lakes to the storks. We also interpolated a raster surface from lake importance using an inverse distance weighted (IDW) technique to illustrate stork distribution (Tang and Yang 2006). To evaluate effects in flock size between removed and net pens wetlands, we fitted a linear mixed model (LMM) to each flock size, which allows the inclusion of a random term that can be used to account for non‐independence between some samples.

Before the analysis, we divided four levels to characterize flock size: 3–10, 11–20, 21–30, and > 30 individuals. We divided four levels because the foraging activity levels of a focal individual had relatively significant differences in four levels during most of the time of an observation session (> 90%, preliminary observation). For the analysis of the activity time budget at different levels of flock size in the PP and NPP (representing before and after the removal of the net pens, respectively), we used the nonparametric Kruskal‐Wallis tests. For each focal individual, we recorded the number of fish caught and sympatric waders. Additionally, we also calculated the rate of foraging in each focus sample. Finally, we examined the differences in foraging rate, number of fish caught, and number of waders at different levels of flock size using non‐parametric Kruskal–Wallis tests.

Then, we used three variables to characterize foraging levels: foraging rate (the total number of pecking within a one‐min period), foraging effort (the ratio of the total amount of time spent searching for and processing food and the activity time budget), and foraging success rate (the percentage of times that the foraging was successful as a percentage of the total number of foraging behaviors). For the analysis, we examined the effects of year, flock size, and water level on the three variables that characterize foraging levels using a linear mixed model. In running these models, we used year as a fixed factor, and flock size and water level as covariate factors.

All statistical analyses were performed with R version 3.4.4 and SPSS 22.0. A significance level of (*p*) 0.05 was used for all statistical tests, with results stated as Mean ± SE.

## 3 Results

### 3.1 Spatial Distribution Patterns

**FIGURE 3:**
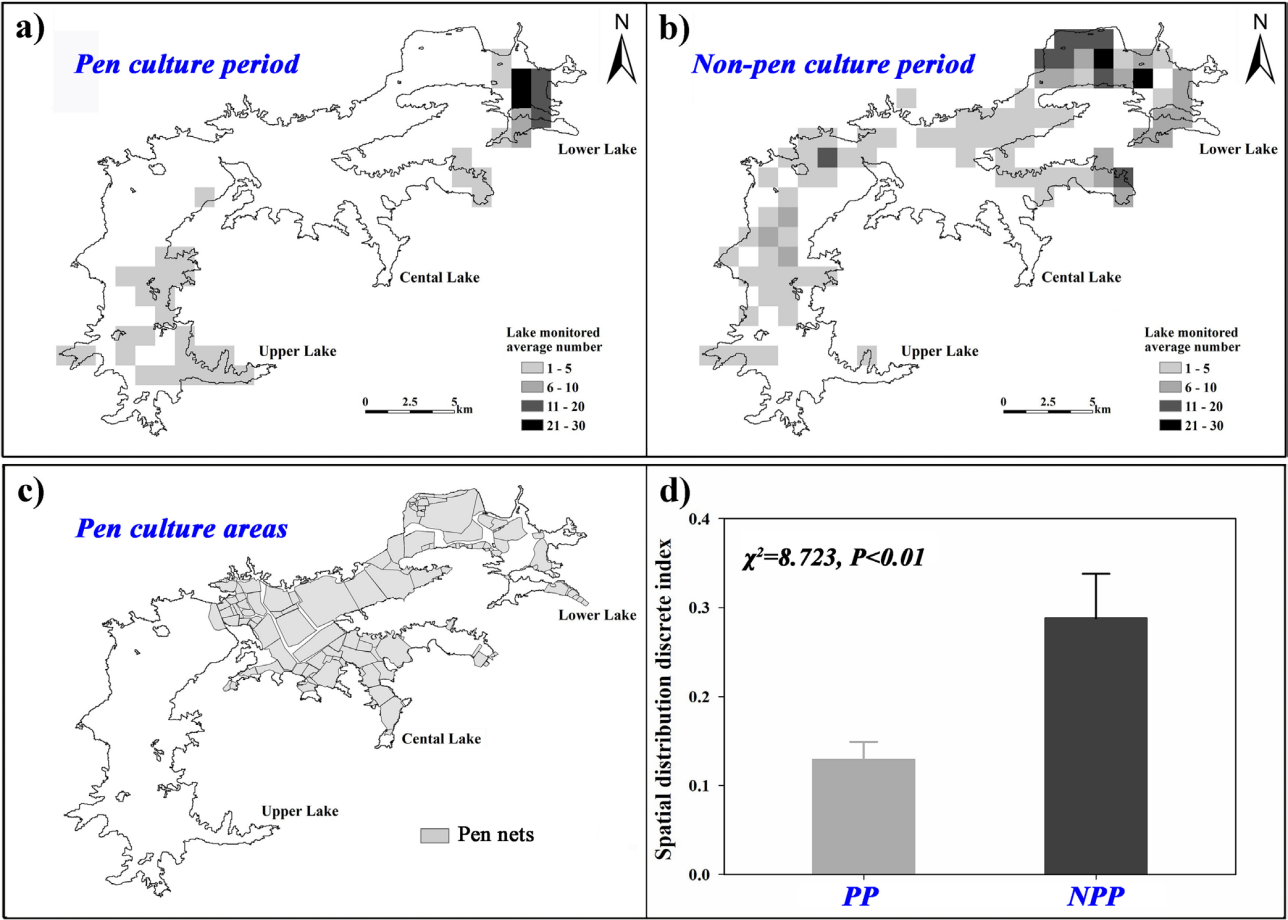
Illustration the change of the spatial distribution and abundance of the wintering Oriental Storks at Shengjin Lake using the Inverse Distance Weighted (IDW) technique. (a) Pen culture period (PP), (b) non‐pen culture period (NPP), (c) pen culture areas in 2017 winter, and (d) spatial distribution discrete index in PP and NPP of Shengjin Lake.

Our results indicated that the spatial distribution pattern of wintering storks grew more dispersed, and the range of their activities further expanded after the net pens were all removed (Figure 3). In PP, the storks' population was generally distributed in the upper lake and lower lake. However, no individuals were observed in the central lake, where deep water dominated during the entire wintering period and extensive net pens. The maximum population number observed of the wintering storks was 131, and the average number was 13.15 ± 0.57 ind. in the grid. In NPP, the storks' population distribution range expanded; among this population, besides the upper and lower lake, the central lake also provided the most suitable foraging habitats for the wintering storks. The maximum population number sharply increased to 407, and the average number increased to 33.07 ± 0.75 ind. at the same time.

**FIGURE 4:**
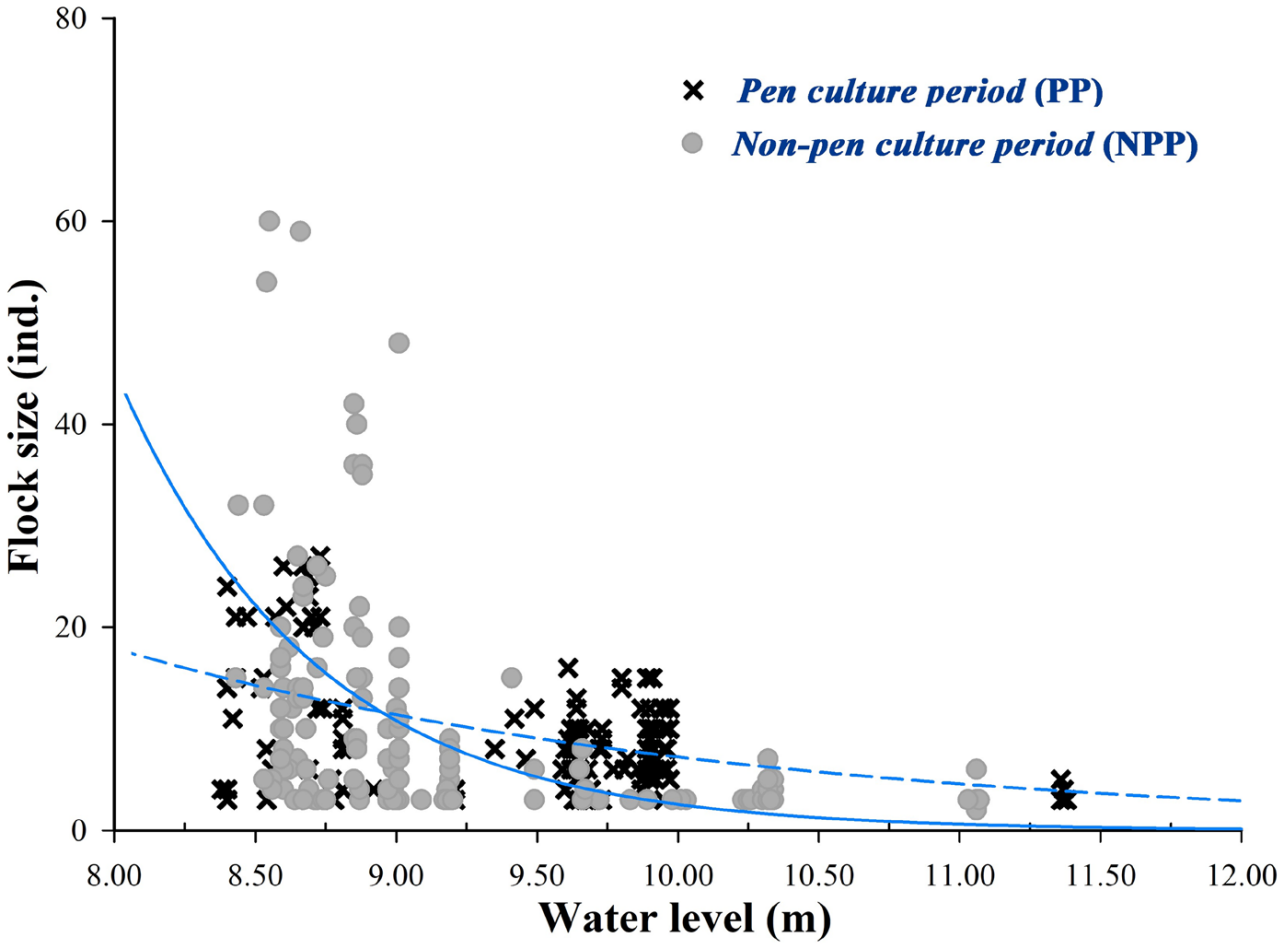
Flock size as a function of water level in Oriental Storks wintering at Shengjin Lake during pen culture period (PP) and non‐pen culture period (NPP). Each point represents a single sample, and the two periods fitted regression lines are shown as dashed (pen culture period) and solid (non‐pen culture period) trend lines, respectively.

LMM showed a significant difference in flock size of storks between PP and NPP (*F*_1,415_ = 270.354, *p* < 0.001), with NPP having a higher spatial distribution discrete index (0.288 ± 0.031 > 0.129 ± 0.013; *χ* ^2^ = 8.723, *p* < 0.01) (Figure 4 & Figure 3d). Flock size decreased linearly with water level (*F*_1,415_ = 132.685, *p* < 0.001). The linear decrease in flock size was more pronounced in NPP (*β* ± SE = 45.34 ± 2.8, *p* < 0.001) than in PP (*β* ± SE = 18.20 ± 2.2, *p* < 0.001) (Figure 4). In the high water level period, the storks showed a multiple small flock size pattern scattered across the distribution at the outer lake. When the water level fell to a low level, storks concentrated in shallow water areas were larger in flock size and tended to distribute inside the lake area.

### 3.2 Effects of Flock Size on Activity Time Budget

Wintering storks allocated the majority of their time to foraging, alert, locomotion, social, and maintenance behaviors. Time spent foraging represented the largest component of the time budget for a different levels of flock size of storks in the two winters (Table 1). The storks spent more time foraging in NPP than in PP (47.20% ± 0.60% > 34.74% ± 1.63%, *χ* ^2^ = 46.467, *p* < 0.001). When flock size is relatively small (< 10 ind.), storks spent less time foraging than the larger group in PP. The storks spent more time alert in PP (0.79% ± 0.24%) than in NPP (0.51% ± 0.11%). Small stork flocks spent more time alert than individuals living in a larger group in the two winters (1.16% ± 0.42%, 2.93% ± 1.66%). Furthermore, alerts were highest in small scale flocks, while PP was significantly higher than in NPP (*χ* ^2^ = 1.091, *p* = 0.779). However, the time allocation of locomotion in NPP (27.97% ± 0.45%) was higher than that of PP (10.00% ± 0.75%). Among different flock sizes, the medium scale was higher than other sizes for time allocation on locomotion in both NPP and PP. The percentage of time spent on resting, social, and maintenance was significantly influenced by year (*χ* ^2^ = 132.765, *p* < 0.001; *χ* ^2^ = 177.999, *p* < 0.001; *χ* ^2^ = 60.174, *p* < 0.001). The storks spent more time on resting and maintenance in PP (31.60% ± 1.33%, 19.85% ± 1.27%) than in NPP (15.41% ± 0.54%, 4.79% ± 0.19%), but social behavior showed the opposite trend (3.03% ± 0.64% < 4.14% ± 0.20%).

**TABLE 1:** Activity time budget of the wintering Oriental storks of different flock size levels in the pen culture period (PP) and non‐pen culture period (NPP) of Shengjin Lake.

Flock size levels (Ind.)	Average flock size (Ind.)	Activity time budget (%)					
		Resting	Locomotion	Alert	Foraging	Social	Maintenance
Pen culture period (PP)							
3–10	5.43 ± 0.18	34.64 ± 2.18	9.25 ± 1.25	1.16 ± 0.42	27.67 ± 2.54	3.91 ± 1.09	23.37 ± 2.13
11–20	15.40 ± 0.40	30.27 ± 2.55	12.57 ± 1.35	0.17 ± 0.13	41.57 ± 2.98	2.78 ± 1.12	12.26 ± 1.66
21–30	25.68 ± 0.39	25.81 ± 1.28	9.31 ± 0.90	0.64 ± 0.45	44.21 ± 2.14	1.35 ± 0.60	18.68 ± 1.67
> 30	34.85 ± 0.72	29.69 ± 3.37	8.10 ± 1.73	0.11 ± 0.02	39.59 ± 6.44	1.56 ± 0.72	20.95 ± 3.84
	*p* < 0.001	*p* = 0.193	*p* < 0.001	*p* = 0.779	*p* < 0.001	*p* = 0.027	*p* = 0.07
Non‐pen culture period (NPP)							
3–10	6.13 ± 0.479	22.88 ± 6.82	18.75 ± 3.61	2.93 ± 1.66	44.98 ± 4.67	0.11 ± 0.01	0.46 ± 0.21
11–20	17.10 ± 0.23	18.09 ± 1.11	29.45 ± 0.76	0.24 ± 0.06	43.66 ± 1.22	3.24 ± 0.48	5.32 ± 0.55
21–30	25.96 ± 0.25	14.09 ± 0.53	25.69 ± 0.77	0.26 ± 0.58	50.21 ± 0.89	4.97 ± 0.32	4.87 ± 0.21
> 30	45.62 ± 0.62	13.82 ± 0.48	29.05 ± 0.58	0.45 ± 0.05	47.20 ± 0.74	4.51 ± 0.30	4.98 ± 0.26
	*p* < 0.001	*p* < 0.01	*p* < 0.01	*p* < 0.001	*p* < 0.001	*p* < 0.001	*p* < 0.001
PP	13.15 ± 0.57	31.60 ± 1.33	10.00 ± 0.75	0.79 ± 0.24	34.74 ± 1.63	3.03 ± 0.64	19.85 ± 1.27
NPP	33.07 ± 0.75	15.41 ± 0.54	27.97 ± 0.45	0.51 ± 0.11	47.20 ± 0.60	4.13 ± 0.20	4.79 ± 0.19
	*p* < 0.001	*p* < 0.001	*p* < 0.001	*p* < 0.001	*p* < 0.001	*p* < 0.001	*p* < 0.001

### 3.3 Effects of Flock Size on Foraging Efficiency

In total, 243 successful foraging bouts were recorded in storks during two winters in 2017 to 2018. Among these, 113 bouts were observed in PP, and 130 bouts were observed in NPP. The storks preferred fish with shorter body sizes (< 10 cm), which accounted for 62.83% and 53.85% of the number of annual fish caught, respectively. Furthermore, the flock size of storks was 21–30 when the storks caught the most fish, and we recorded 44 occurrences and 57 occurrences in the two winters (Table 2). Nevertheless, when the flock size of storks was too small or large, the number of fish caught was both relatively less.

**TABLE 2:** The number of fish with their size taken by the wintering Oriental Storks in the pen culture period (PP) and non‐pen culture period (NPP) of Shengjin Lake.

Fish size	Pen culture period (PP)					Non‐pen culture period (NPP)				
	Flock size (Ind.)					Flock size (Ind.)				
	3–10	11–20	21–30	> 30	Total	3–10	11–20	21–30	> 30	Total
< 10 cm	7	29	26	9	71	12	16	32	10	70
10–20 cm	4	9	15	6	34	6	13	21	7	47
> 20 cm	2	3	3	1	8	4	2	4	3	13
Total	13	33	44	23	113	22	31	57	20	130

The foraging rate of storks varied at different flock sizes in PP; the times of jabbed water in search of food (12.98 ± 2.26 Pecks/min) and the number of fish caught (3.27 ± 0.33 Ind./min) were the highest when flock size was 21–30 compared to other levels. In NPP, the foraging rate was the highest when flock size was over 30 individuals (35.39 ± 2.27 Pecks/min) and significantly different from other levels (*χ* ^2^ = 16.566, *p* < 0.001). Same as the PP, the number of fish caught (4.57 ± 0.72 Ind./min) was the highest when flock size was 21–30 compared to other levels. When two periods of data were compared together the foraging rate in NPP was significantly higher than in PP (*χ* ^2^ = 41.200, *p* < 0.001). However, the number of fish caught did not vary between years (*χ* ^2^ = 2.281, *p* = 0.131) (Table 3).

**TABLE 3:** The number of fish caught and the foraging rate of Oriental Storks in the presence of other fish‐eating waterbirds in the pen culture period (PP) and non‐pen culture period (NPP) of Shengjin Lake.

Flock size levels (Ind.)	Foraging rate (Pecks/min)	No. of fish caught (Ind.)	No. of waders (Ind.)
Pen culture period (PP)			
3–10	9.90 ± 1.87	2.54 ± 0.49	20.00 ± 5.97
11–20	11.17 ± 2.28	2.67 ± 0.33	52.36 ± 14.94
21–30	12.98 ± 2.26	3.27 ± 0.33	17.17 ± 6.29
> 30	8.76 ± 1.79	1.80 ± 0.20	25.90 ± 4.97
	*χ* ^2^ = 1.817	*χ* ^2^ = 6.098	*χ* ^2^ = 7.078
	*p* = 0.611	*p* = 0.107	*p* = 0.069
Non‐pen culture period (NPP)			
3–10	17.29 ± 1.23	2.36 ± 0.32	12.21 ± 3.06
11–20	28.28 ± 4.19	4.13 ± 0.64	58.14 ± 17.79
21–30	31.36 ± 2.25	4.57 ± 0.72	60.63 ± 12.69
> 30	35.39 ± 2.27	3.75 ± 0.95	43.50 ± 12.43
	*χ* ^2^ = 16.566	*χ* ^2^ = 9.285	*χ* ^2^ = 16.935
	*p* = 0.001	*p* = 0.026	*p* = 0.001
PP	9.44 ± 0.93	2.67 ± 0.21	28.89 ± 5.37
NPP	24.04 ± 1.50	3.25 ± 0.27	32.35 ± 5.52
	*χ* ^2^ = 41.200	*χ* ^2^ = 2.281	*χ* ^2^ = 0.595
	*p* < 0.001	*p* = 0.131	*p* = 0.441

The mixed linear model results indicated that the peaking rate of storks in NPP was significantly higher than PP (coeff. ± SE = 10.51 ± 2.18, *t* = 4.81, *p* < 0.001), while water level (*t* = 1.57, *p* = 0.121) and flock size (*F* = 1.66, *p* = 0.202) had no effect. In terms of foraging effort, pen culture (*t* = 1.63, *p* = 0.108), water level (*t* = 0.25, *p* = 0.807) and flock size (*t* = 0.19, *p* = 0.851) had no significant impact. Furthermore, the foraging success rate of storks in NPP was significantly lower than PP (coeff. ± SE = −0.10 ± 0.02, *t* = −5.11, *p* < 0.001), but not affected by flock size (*F* = 0.14, *p* = 0.706) (Table 4).

**TABLE 4:** Effects of year, water level, and flock size on foraging of Oriental Storks in the pen culture period (PP) and non‐pen culture period (NPP) of Shengjin Lake.

	Parameter	Estimate	SE	*t*	*p*	LCL	UCL
Foraging rate	Intercept	25.50	9.37	2.72	0.008	6.760	44.238
	Pen culture^a^						
	*PP*	10.51	2.18	4.81	0.000	6.138	14.876
	Water level	−1.60	1.02	1.57	0.121	3.647	0.438
	Flock size	−0.07	0.05	1.29	0.202	0.172	0.037
Foraging effort	Intercept	0.63	0.23	2.75	0.008	0.173	1.092
	Pen culture^a^						
	*PP*	0.09	0.05	1.63	0.108	0.020	0.195
	Water level	0.01	0.03	0.25	0.807	0.044	0.056
	Flock size	0.00	0.00	0.19	0.851	0.002	0.003
Foraging success rate	Intercept	−0.05	0.09	0.63	0.530	0.224	0.116
	Pen culture^a^						
	*PP*	−0.10	0.02	5.11	0.000	0.141	0.062
	Water level	−0.03	0.01	3.40	0.001	0.020	0.010
	Flock size	0.00	0.00	0.38	0.706	0.001	0.001

^a^ The reference category for period is “non‐pen culture (NPP).”

## 4 Discussions

### 4.1 Strategic Adjustment of Activity Time Budget

Our study revealed that the composition of the activity time budget changes with flock sizes of the wintering storks. Specifically, we found the time of foraging and locomotion increased to some extent with larger group sizes to obtain abundant food resources. The first hypothesis was confirmed. As a strategy, allocating more time and budget to foraging increases the chances that individuals will feed in more abundant and available areas (Coulombe et al. 2008). However, the time allocated to foraging decreases with flock size beyond a certain threshold, due to increased competition among species, which leads to the rapid depletion of food resources in larger groups. Storks are large carnivores with strong beaks and aggressive in nature, so they do not need to spend as much time alert individually or in groups (Shao et al. 2015). Nonetheless, storks would spend relatively more time on the alert in small groups because small groups are scattered around the lake, shortening the distance from the edge of activity areas to the human settlements (Li et al. 2014). When the water level declined, the storks began to shift their location from the human activity areas to the center of the lake. The relatively safer environment partly allows them a reduction of alerts (Li et al. 2015).

Mwanwhile, we found a significant difference in foraging, resting, locomotion, and maintenance behaviors of storks before and after the removal of the net pens. Storks spent more time foraging and locomoting in NPP than in PP, which might be the fragmented habitats that were reconnected and recovered. Furthermore, the increased movement capacity of fish in the non‐pen culture areas (Wang et al. 2019) forced the storks to increase their locomotion time budget to improve the chances of catching fish in NPP. After the net pens were removed and artificial fishery farming activities completely stopped, the disturbance levels of human activities were significantly decreased; the storks increased the time budget for foraging to obtain sufficient food resources, which could compensate for energy costs during the wintering period. In addition, the time budget for alerts in the storks differed significantly between NPP and PP. In PP, the storks would allocate more time on alert, suggesting frequent artificial fishery farming activities, increasing the predation risk of waterbirds.

### 4.2 Spatio‐Temporal Dynamics of Flock Distribution and Size Patterns

Our results showed that the spatial distribution range of storks significantly expanded after the removal of net pens, confirming the second hypothesis. A few storks have been recorded sporadically in the eastern part of the upper lake where the Zhangxi River flows into the lake in PP. Meanwhile, no storks were found in the middle lake, which was covered by a large area of net pens during the winter of 2017. This result might be a consequence of cumulative impacts from pen culture. Different habitat changes triggered by pen culture, such as habitat fragmentation, human activities, and intensified competition (Fang et al. 2006; Haddad et al. 2015). Moreover, the net pens divided Shengjin Lake into discrete sections, which not only limit the movement and dispersal of fish species (Wu et al. 2001; Jia et al. 2013) but also the connectivity of the foraging habitats of the storks. Fish stocked in pens grew well using the natural food present, with aquatic macrophytes being abundant (Jia et al. 2013). The massive increase in larger cultured species could lead to a significant decline in the populations of small wild fish (Machias et al. 2004; Ding et al. 2016), making it more difficult for storks to catch suitable food in pen culture areas. In addition, fish migrations among different habitat patches can be disrupted in fragmented habitats, leading to homogenization of species compositions between communities and further decreasing the availability of food resources.

Habitat availability of waterbirds in seasonally recessional wetlands is highly linked to water level regimes, which vary with seasonal flooding, resulting in spatio‐temporal heterogeneity in water depth (Baschuk et al. 2012; Li et al. 2019). As fitted regression, the flock size of storks was largely correlated with the water level changes at Shengjin Lake in our study (Figure 4). Wintering storks were mainly distributed in small flocks, where the upper part of the lake and shallow water areas were at the edge of Shengjin Lake during high water level periods. With the progress of the wintering period, water began to recede, and the diversity of shallow water and mudflat habitats were gradually exposed, providing foraging grounds for a large number of waterbirds in different foraging guilds (Holm and Clausen 2006; Zhang et al. 2021). When the water level decreased, many storks gathered in those shallow water areas to increase the chance of obtaining food, such as the sites of Yanwo, Waipai, and Gangyao in the lower part of the lake. Due to the relatively higher elevation of those areas, the food was exposed first, and it lasted for a longer duration (Li et al. 2019). Meanwhile, variations in the lakebed topography caused small fish to congregate in shallow waters, whereas larger fish were restricted to deeper waters, leading to resource segregation that influenced the spatial distribution of storks. Although concentrated in the shallow water foraging brought competitive pressure, foraging attempts and capture successes were significantly higher for waterbirds foraging in groups than for those foraging in group break‐ups or individually (Cheng et al. 2020). This may be another reason for the gathering of storks in lower water level areas.

### 4.3 Modeling of Foraging Efficiency Is Affected by Ecological Factors

The model results clarified that the pen culture had a significant effect on the foraging activity level of the wintering storks. We found a higher foraging rate and number of fish caught by storks among the study area in NPP than that in PP. Nevertheless, the foraging success rate in NPP showed a significantly lower average level than that in PP. The differences in foraging activity were not only incurred by food availability, abundance, and distribution, but also by fishery patterns and fish size. In PP, the presence of net pens limited the movement and dispersal of fish species (Wu et al. 2001; Jia et al. 2013). In addition, net pens trapped large fish in pen culture areas and excluded small wild fish to non‐pen culture areas (Machias et al. 2004). This may explain the higher food capture and foraging success rate for storks in PP of Shengjin lake. Even if aquaculture could play a complementary role when food was scarce, it was difficult to compensate for the lower food availability in PP. Therefore, storks might adopt a conservative foraging strategy by reducing the foraging rate in PP, as this could lower the energy cost associated with frequently lowering their heads to search for food in the water. In addition, a low foraging rate may have led to longer time gaps between food searches when relying on visual techniques. In contrast, the storks adopted a relatively radical foraging strategy by increasing their foraging rate in NPP. This may be due to the fish reducing their risk of predation by more flexible mobility after the demolition of net pens; the storks improved the pecking rate to increase chances for food by tactile techniques.

A wading bird can select the most appropriate foraging behavior for its needs, and the choice of a successful foraging behavior should reinforce repeated use (Kushlan 1978). Thus, the selection of a specific foraging technique among fish‐eating waterbirds depends on the condition and quality of wetlands as affected by fluctuating water levels (Maheswaran and Rahmani 2001). In our study, storks showed higher foraging success during low water levels and lower success during high water level periods. It should be noted that the availability increase of prey incurred by water level recession and habitat connectivity was the primary driver for the variations of foraging success rates in NPP. Moreover, when foraging at a low water level, the turbidity of the water body increased, and the storks adjusted to tactile mode and improved the frequency of foraging (Maheswaran and Rahmani 2001). Kushlan's (1978) hypothesis suggests that, in the context of food selection, birds that primarily search for food should be specialists, whereas birds that either actively search for or passively wait for food to approach them are likely to be more generalized. Consequently, the storks were gradually shifting from specialists to opportunists in response to the changing external conditions of wetlands. Meanwhile, Kushlan (1978) also reported that the water oxygen content varies with water body size and has an effect on fish behavior. This would result in low water level periods where the dissolved oxygen content of the water body was reduced and fish shifted to shallow water or close to the surface for gulping air, which makes them more susceptible to capture by storks.

## 5 Conclusions

Taken together, this study demonstrated significant negative impacts of pen culture on spatial distribution and activity patterns for the wintering storks at Shengjin Lake. Pen culture resulted in an overall loss of spatial distribution and a reduction in foraging efficiency of storks due to habitat alteration and fragmentation in PP. Moreover, a more flexible and radical trend in the foraging patterns of the wintering storks was triggered by the combined effects of the net pens removal and habitat connectivity in NPP. Our study highlights the urgent need to carry out assessments of fishery on wetland, which may lead to increased ecological risks for waterbirds in floodplain lakes of the Yangtze River. Meanwhile, we have provided a small scale but important case study, adding to our knowledge of Oriental Storks in aquaculture fisheries in the Yangtze River floodplain. The results have implications for the conservation management of other endangered waterbirds facing pressures from culture‐based fisheries.

## Author Contributions

**Lei Cheng:** conceptualization (equal), data curation (lead), formal analysis (equal), investigation (lead), methodology (equal), software (lead), visualization (lead), writing – original draft (lead), writing – review and editing (lead). **Lizhi Zhou:** conceptualization (lead), formal analysis (lead), funding acquisition (lead), project administration (lead), resources (lead), supervision (lead), validation (equal), visualization (lead), writing – original draft (equal), writing – review and editing (equal). **Chao Yu:** investigation (supporting), methodology (supporting), writing – original draft (supporting), writing – review and editing (supporting). **Yiwei Bao:** investigation (supporting), methodology (supporting), writing – original draft (supporting), writing – review and editing (supporting).

## Ethics Statement

We obtained approval from the reserve management offices before experimenting. All the observations were conducted under the natural status of Oriental Storks and had no influence. All the field observations conducted in this study were performed in accordance with the current laws of China.

## Conflicts of Interest

The authors declare no conflicts of interest.

## Supporting information


Table S1.

## Data Availability

Data are provided as Table S1.
